# The focal adhesion protein Integrin-Linked Kinase (ILK) as an important player in breast cancer pathogenesis

**DOI:** 10.1080/19336918.2020.1829263

**Published:** 2020-10-12

**Authors:** Katerina Tsirtsaki, Vasiliki Gkretsi

**Affiliations:** Department of Life Sciences, School of Sciences, European University Cyprus, Nicosia, Cyprus

**Keywords:** Breast cancer, metastasis, invasion, ILK, PINCH-1, PARVA, Rictor, Akt, EMT, focal adhesions

## Abstract

Cell-extracellular matrix interactions, or focal adhesions (FA), are crucial for tissue homeostasis but are also implicated in cancer. Integrin-Linked Kinase (ILK) is an abundantly expressed FA protein involved in multiple signaling pathways. Here, we reviewed the current literature on the role of ILK in breast cancer (BC). Articles included in vitro and in vivo experiments as well as studies in human BC samples. ILK attenuation via silencing or pharmaceutical inhibition, leads to apoptosis or inhibition of epithelial-to-mesenchymal transition, and cell invasion whereas ILK overexpression suppresses anoikis and promotes tumor growth and metastasis. Finally, ILK is upregulated in BC tumors and its expression is associated with grade, and metastasis. Therefore, ILK should be evaluated as a potential anti-cancer pharmaceutical target.

Breast cancer (BC) is the most common cancer type among women worldwide being responsible for the majority of cancer-related mortality in women [1]. There are various types of BC depending on the type of cells the tumor originates from, the tumor size, the degree of cell differentiation [2,3], the malignancy grade, and the tendency of the cells to invade through surrounding tissue [4].

## Cell-matrix adhesions and Integrin-Linked Kinase (ILK)

Cell-extracellular matrix (ECM) interactions, also known as focal adhesions (FA), are of fundamental importance in normal tissue homeostasis but they are also critically involved in cancer progression and metastasis [5]. Integrin-Linked Kinase (ILK) is an abundantly expressed FA protein implicated in many cellular processes and signal transduction pathways that control cell survival, differentiation, proliferation, and gene expression in mammalian cells, while it has also been associated with certain pathological conditions [6,7]. In fact, increased ILK expression has been correlated with cancer progression in several cancer types, rendering ILK a potential anti-cancer therapeutic target [8].

ILK comprises three major domains: an Ν-terminal domain that contains four ankyrin repeats, a central pleckstrin homology (PH)-like domain, and a C-terminal kinase domain (KD). ILK is also known to serve as an adaptor protein at FA sites, where it interacts with multiple other proteins and regulates normal cellular functions [9]. The N-terminal domain has been shown to bind to the FA protein Particularly Interesting New Cysteine Histidine protein (PINCH-1). The same domain also binds to ILK-associated protein (ILKAP), a protein phosphatase 2 C (PP2C) that negatively regulates ILK signaling [10]. Adjacent to the ankyrin repeats, a sequence motif present in PH domains binds to the second messenger phosphatidylinositol 3,4,5-trisphosphate (PIP3) and a phosphoinositide 3-kinase (ΡΙ3 K)-dependent kinase activation has been reported [11]. The C-terminal kinase domain also interacts with β1-integrin [12], as well as with the FA proteins paxillin [13] and parvins [9,12,14], which link ILK, and therefore integrins, to the actin cytoskeleton.

## ILK-mediated signaling

ILK was originally considered to function as a serine/threonine kinase at FAs. Being stimulated by integrins and soluble mediators, including growth factors and chemokines, it was shown to be regulated in a PI3K-dependent manner [15,16]. Moreover, the activity of ILK was demonstrated to be antagonized by phosphatases such as ILKAP and phosphatase and tensin homolog (PTEN) [17,18].

**Figure 1.**
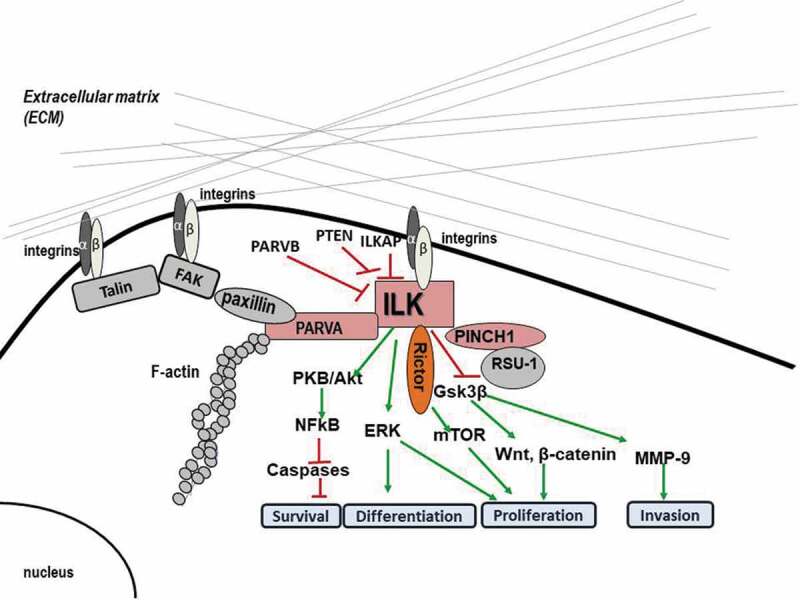
Diagram depicting the main molecular interactions of ILK at FAs as well as the major signaling pathways involved.

Important downstream targets of ILK signaling (as shown in Figure 1) include pro-survival pathways such as PKB/Akt, glycogen synthase kinase 3 beta (GSK-3β), β-catenin, p44/p42 MAP kinases, the myosin light chain (MLC) [19], and the Hippo pathway [20]. The PKB/Akt pathway is a crucial regulator of cell survival and apoptosis. In fact, to become fully activated, PKB/Akt requires phosphorylation at two sites, threonine 308 and serine 473 which mainly depends on PI3K activity, the rictor-rapamycin complex 2 (mTOR) complex [21], and the ILK-Rictor complex [10]. Interestingly, yeast two-hybrid assay demonstrated a direct interaction between the ΝΗ_2_- and COOH-terminal domains of Rictor and the ILK KD [22]. Rictor appears to have similar functions to ILK being a regulator of cytoskeletal dynamics, and a component of the mammalian target of mTORC2, a complex implicated in Akt phosphorylation. GSK-3β is phosphorylated and inactivated at serine 9 by ILK, regulating the cell cycle through proteolysis of cyclin D1 and activation of the transcription factor Activator protein 1 (AP1) [23]. Inactivation of GSK-3β, in turn, stabilizes β-catenin, whose accumulation is related to deregulation of proliferation, migration, and differentiation [24] while ILK can also directly phosphorylate MLC on Ser18/thr19 affecting cell contraction, motility, and migration [25].

However, the initial study of the sequence of the KD had raised suspicions that ILK might have been a pseudokinase [26]. Although the ILK KD contains a few domains commonly encountered in kinases such as the lysine residue in subdomain II that is necessary for phosphotransfer, the glutamic acid in subdomain III involved in ATP binding and the A/SPE (Ala/Ser-Pro-Glu) motif in subdomain VIII required for substrate recognition, it also contains a domain that does not fit with what is known for kinases. More specifically, the ATP-binding P loop in subdomain I of the KD contains a non-flexible non-glycine-rich NENHSG (AsnGlu-Asn-His-Ser-Gly) motif [27] instead of a flexible one. Most importantly, the ILK KD does not contain the invariant catalytic base aspartate residue in the HRD (His-Arg-Asp) motif in subdomain VIb, which is responsible for accepting a proton from the hydroxyl group of the substrate during the phosphotransfer reaction. Moreover, in ILK, there is a substitution of the DFG (Asp-Phe-Gly) motif in subdomain VII activation loop for DVK (Asp-Val-Lys) which is also indicative of ILK being a pseudokinase, as several kinases lacking the GXGXXG motif or the DFG and APE motifs are thought to be atypical and are generally shown to be inactive [28].

## ILK as an adaptor protein and the ILK-PINCH-PARVA (IPP) complex

Interestingly, many studies have also demonstrated that ILK plays a significant role at FAs by serving as an adaptor protein participating in multiple protein–protein interactions. In fact, this very fact could also provide an explanation as to how ILK transduces signals if it is not a true kinase but rather a pseudokinase. Specifically, ILK has been shown to form a stable ternary complex at FA sites by binding to PINCH-1 and alpha-parvin (PARVA) (Figure 1). This protein complex known as IPP (ILK-PINCH-parvin) localizes to FAs and is essential for several integrin-dependent functions [9]. The IPP complex interacts with the cytoplasmic tail of β1 integrin, resulting in the engagement and organization of the cytoskeleton as well as activation of signaling pathways. The significance of these proteins for FAs is evidenced by experiments involving depletion of *ILK* or *PINCH-1* genes which clearly showed that maturation of FAs is blocked through down-regulation of the expression or reduction of the recruitment of tensin and destabilization of α5β1-integrin-cytoskeleton linkages [29]. Of course, the formation of the IPP complex is not the only protein–protein interaction at FAs. In fact, there are multiple such interactions at FAs which actually form what is known as the cell’s adhesome [30].

Thus, apart from its connection to PINCH-1 and PARVA, ILK is indirectly connected to multiple other proteins which in turn transduce signals to several other signaling pathways. For instance, binding of PINCH-1 to Nck-2 connects it with Insulin Receptor Substrate-1 (IRS-1) which is in turn associated with ligand-activated growth factor receptors such as Platelet-Derived Growth Factor (PDGF) receptor β [6]. Also, PINCH-1 interacts with Ras Suppressor-1 (RSU1) [31] being connected to Ras-mediated signaling.

Hence, ILK through its interactors has been postulated to contribute to the formation of supramolecular complexes containing both components of the FAs and components of signaling pathways [6].

The aim of this literature review is to apprehend the function of ILK in BC with a view to use it in identifying better therapeutic approaches to deal with the disease and ultimately restrict BC formation and metastasis. A summary of the major findings of each study is shown in Table 1.

**Table 1.** Summary of studies investigating the role of ILK in BC using *in vitro*, and *in vivo* models as well as human patient samples.

Study	*In vitro*	*In vivo*	Human BC samples	Experimental design	Functional effect	Signaling
[32]	√			Overexpression of ILK in MDA-MB-231 and MDA-MB-435 BC cells	Suppression of anoikis which is reversible by transfection of dominant negative kinase dead ILK	
[47]	√			Inhibition of ILK by QLT-0267 in MDA-MB-231 cells	BC cell apoptosis	Reduced mTOR expression and PKB/Akt Ser473 phosphorylation
[44]	√			ILK attenuation either via silencing or through pharmacological inhibition	ILK overexpression promotes cell migration	-Hyperphosphorylation of ERα
[48]	√			ILK attenuation either via silencing or through pharmacological inhibition in MDA-MB-231 cells	-Inhibition of cell invasion	-Blocking of Akt, ERK, c-Jun, and uPA.
[22]	√			Depletion of ILK and Rictor in MDA-MB-231 cells	BC cell apoptosis	-ILK binds to Rictor -Inhibition of Akt Ser473 phosphorylation
[51]	√			-ILK overexpression -ILK inhibition by QLT-0267 -ILK silencing in 6 BC cell lines (LCC6^Her2^, MCF7^Her2^, SKBR3, ΒΤ474, JIMT-1, KPL-4)		ILK regulates Akt Ser473 phosphorylation, YB-1 expression and promoter activity, and Twist expression.
[52]	√			-Suppression of ILK by ILK inhibitor T315 or gene silencing in MDA-MB-468 cells -Rictor silencing		- ILK or Rictor silencing inhibits phosphorylation of Ser473-Akt
[35]	√			-ILK inhibition in BC cells MCF10A and MDA-MB-231 cells	- Suppression of ILK suppresses EMT	ILK suppresses Hippo pathway (MST1, LATS1) and promotes YAP/TAZ
[40]	√			-Overexpression of Twist or integrin β1 in MCF10A breast epithelial cells and TRAQ-labeling combined with 2D LC-MS/MS analysis. -Twist, ILK, or integrin β1 silencing in BT549 and Hs578T	-Twist or integrin β1 silencing reduces ILK and impairs EMT and cell invasion. -ILK silencing suppresses Twist mediated EMT and invasion	-integrin β1 or Twist overexpression regulates ILK
[16]	√			-ILK overexpression in MCF-7 cells -ILK silencing in MDA-MB-231 cells	-ILK overexpression results in cell growth and proliferation.	-Through PI3K/Akt pathway
[57]		√		Transgenic mice overexpressing ILK under the MMTV promoter	Tumorigenicity and tumor hyperplasia.	-Induction of PKB/Akt, GSK-3β and ERK phosphorylation.
[60]	√	√		-Overexpression of ILK in MDA-MB-435 cells (ILK deficient) and in vivo in athymic nude mice	-Reduction in proliferation, migration, and tumor formation and metastasis in nude mice. -ILK is downregulated in metastatic BC cells →ILK deficiency facilitates neoplastic growth and metastasis	-Through its ability to block cell cycle progression in G1 phase by blocking integrins
[49]	√	√		- Use of ILK inhibitor QLT0267 alone or in combination with chemotherapy drugs. -In vitro in 7 BC cell lines ((LCC6, LCC6^Her2^, SKBR-3, KPL-4, BT-474, MBA-MB-468, and MCF-7) -In vivo using orthotopic xenografts from low Her2-expressing cells (LCC6)	-Docetaxel had synergistic action with QLT0267 resulting in increased cytotoxicity and improved therapy. Other chemotherapy drugs had antagonistic effects. - increased survival in the three models and reduction in the growth of cancer cells.	-Through PI3K/Akt pathway
[39]	√	√		-Conditional ILK knock out mice from the mammary epithelium -Inhibition of ILK by an ILK inhibitor or siRNA-mediated silencing in ErbB2-expressing primary mammary gland cells	-Delay in tumor growth -Induction of apoptosis and reduced cell invasion	
[38]		√		-Transgenic mice expressing both Wnt and ILK in mammary epithelium (under the MMTV promoter)	-Tumor formation and growth is accelerated -Cooperation between ILK and Wnt in BC	Elevated expression of Wnt/ILK targets (beta-catenin and cyclin D1) as well as activation of FOXA1 transcription factor, a marker of differentiated mammary luminal cells.
[55]	√	√		-Ectopic expression or shRNA silencing or pharmacological inhibition (via T315) of ILK in MDA-MB-231, SUM-159, MCF-7, MCF-7-IL6 cells -In vivo effect of T315-induced ILK inhibition on CSCs in SUM-159 xenograft models	- ILK silencing inhibits CSC population in vivo	-ILK regulates IL-6-driven Notch1 activation and CSCs through gamma-secretase components.
[43]	√		√	-Overexpression of PARVB in cell lines MCF-7 and MDA-MB-231 -Gene expression analysis in human samples	-Reduction of cell invasion and anchorage-independent cell growth -Downregulation of PARVB and upregulation of ILK in a significant percentage of BC tumors	-ParvB inhibits ILK and EGF-induced phosphorylation of ILK cellular targets.
[45]	√		√	-Tissue biopsy array consisting of 10 BC biopsy samples	Co-localization of ILK and HIF in human BC samples	
[54]	√		√	- ILK depletion via shRNA-mediated silencing and ectopic expression in MDA-MB-231 cells -ILK depletion in ovo	- In BC samples, ECM stiffness, ILK, and CSC markers (CD44) are associated -Stiff and hypoxic microenvironments promote the development of breast CSC through modulation of ILK. - Depletion of ILK in ovo significantly abrogated the tumorigenic and metastatic potential of invasive BC cells.	-ILK signals through the PI3K/Akt to regulate the development of CSCs
[56]	√		√	-In vitro (MCF-7, MDA-MB-231, MDA-MB-468, SUM-159) 1) IL-6 treatment 2) shRNA or pharmacological inhibition of ILK -In vivo	-ILK attenuation blocks estrogen-independent tumor growth	-IL-6 regulates ILK expression via E2F1 and NFkB to activate again IL-6.
[58]			√	−64 BC samples for real-time PCR -163 BC samples for immunohistochemistry	High ILK expression was correlated with tumor size, grade, stage, ER status, metastasis, and reduced overall survival.	
[59]			√	−96 phyllodes BC	-High ILK expression in the tumor and association with increasing tumor grade.	Analysis of EMT-related genes: -decreased E-cadherin and β-catenin -increased expression of N-cadherin, vimentin, Snail, ZEB1 ,and Twist

## ILK in BC: Epithelial to Mesenchymal Transition (EMT) and Akt phosphorylation

**Figure 2.**
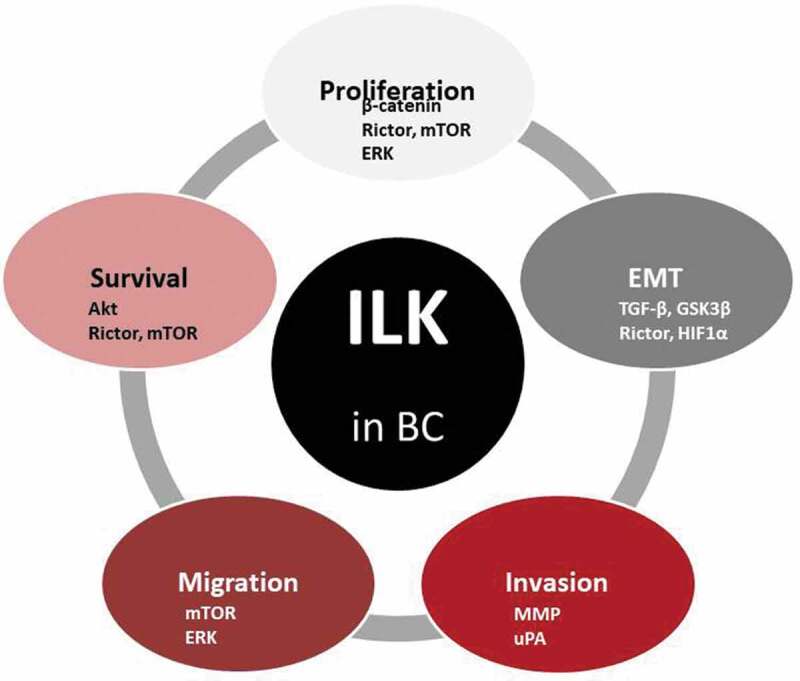
Diagram summarizing the role of ILK in relation to basic cancer properties in BC. The signaling molecules involved are also indicated.

Initially, overexpression experiments demonstrated that ILK suppresses anoikis in MDA-MB-231 and MDA-MB-435 BC cells which is reversible by transfection with a dominant-negative, kinase dead form of ILK [32]. However, it was also reported that ILK’s function is required in Transforming Growth Factor beta (TGFβ-1)-induced Epithelial–Mesenchymal Transition (EMT) in mammary epithelial cells [33,34], promoting a more metastatic phenotype (Figure 2). Moreover, the ILK/Rictor complex has been identified as a potential molecular target for preventing or even reversing this process [35]. Finally, ILK can also directly regulate EMT by promoting the expression of Snail [36] and via posttranslational modifications through GSK-3β.

The latter is of great significance, as the acquisition of invasive and migratory characteristics in cancer cells results primarily from adopting an EMT phenotype. Thus, the fact that overexpression of ILK induces EMT in mammary epithelial cells [37] and its inhibition abolishes *in vitro* cell metastasis [34] corroborates the idea that ILK is a key intracellular mediator of TGFβ-1 induced EMT [33]. Also, a significant acceleration in mammary tumor incidence and growth was observed in the MMTV-Wnt/ILK mice compared to mice expressing Wnt alone, showing the cooperation between Wnt1 and ILK genes during mammary carcinogenesis [38]. Furthermore, mammary epithelial disruption of ILK in mice results in a profound block in mammary tumor induction [39]. Moreover, the inactivation of ILK suppresses Yes-associated protein (YAP) activation and tumor growth *in vivo* [20], indicating that ILK plays a critical role in the suppression of the Hippo pathway in BC cells.

In another study, ILK was also shown to promote EMT and cell invasion in BC cells [40]. Specifically, Yang et al. reported that Twist transcriptionally regulates integrin β1 expression and, in fact, overexpression of one of the two in MCF10A breast epithelial cells makes cells undergo an EMT with concomitant activation of ILK, Extracellular Signal Regulated Kinase (ERK), PI3K/AKT, and WNT signaling while ILK silencing also suppresses EMT and cell invasion [40].

In an *in vitro* study by Qu et al. [16], it was shown that ILK overexpression promotes cell proliferation, while ILK knockdown leads to growth arrest in BC cell lines. In this study, they used two BC cell lines, MCF-7 and MDA-MB-231 cells, and they first tested the endogenous expression of ILK, showing that MDA-MB-231 has much higher ILK expression than MCF-7 cells [16]. They then stably overexpressed ILK in MCF-7 cells by lentivirus transduction which resulted in a significant increase of cell viability through the induction of Akt phosphorylation. At the same time, they silenced *ILK* in MDA-MB-231 cells that had an initially high expression of endogenous ILK. *ILK* silencing conversely inhibited Akt phosphorylation [16]. Conclusively, it was shown that the regulatory effects of ILK on cell growth and proliferation are mainly mediated by PI3K/Akt signaling.

## ILK in BC: its binding partners

In a screen for identifying new ILK binding partners, it was shown that Rictor is one such partner for ILK, and several studies investigated the relationship between ILK and Rictor in BC. Rictor, a component of the mTORC2 complex, which is implicated in Akt phosphorylation, was initially found to be involved in cytoskeleton regulation. Being part of mTORC2, Rictor was shown to regulate the ability of ILK to promote Akt phosphorylation and cancer cell survival [22,41], as depletion of ILK and Rictor from BC cells blocked Akt Ser^473^ phosphorylation and induced apoptosis [22]. Notably, the interaction between ILK and Rictor was also reported by Serrano, et al. [35], where it was shown that TGF-β treatment promoted this interaction. In fact, ILK activity was shown to be essential for ΤGFβ-1-mediated EMT in mammary epithelial cells which also induced expression of the mTOR2 component Rictor and its phosphorylation on Thr1135 [35]. Interestingly, the ILK/Rictor complex formation was promoted more in cancer than in normal cells indicating a true involvement in cancer development. Moreover, inhibition of ILK partially reversed the basal mesenchymal phenotype of ΜDΑ-ΜΒ-231 cells and prevented EMT in MCF10A cells after TGFβ-1 treatment. These data demonstrate a requirement for ILK function in TGFβ-1-induced EMT in mammary epithelial cells and identified the ILK/Rictor complex as a potential molecular target for reversing EMT [35].

Mongroo, et al., (2004) analyzed breast tumors and BC cell lines for the expression of β-parvin (PARVB), another known binding partner of ILK [42]. They clearly demonstrated an inverse relationship between ILK and PARVB expression in BC cells and tissues and further suggested that PARVB inhibits ILK signaling. Specifically, gene expression analysis at the mRNA level demonstrated a significant downregulation of PARVB in four (4) out of nine (9) human breast tumors compared to their patient-matched normal mammary gland tissue, while a dramatic downregulation was also observed at the protein level in five (5) out of seven (7) advanced tumors [43]. Interestingly, ILK protein expression levels were elevated in these tumors. In the same study, PARVB was overexpressed in MCF-7 and MDA-MB-231 BC cells, which originally expressed low levels of PARVB, and its overexpression led to inhibition of ILK kinase activity, anchorage-independent cell growth, and *in vitro* cell invasion in MDA-MB-231 cells accompanied by inhibition of EGF-induced phosphorylation of two ILK targets, PΚΒ (Ser^473^) and Gsk3β (Ser9) [43].

Moreover, ILK has been shown to bind to estrogen receptor-alpha (ERα) both in *in vitro* and *in vivo* studies [44] and, in fact, it seems that ILK signaling is a modulator of ER signaling in BC, with major consequences for BC patients, as ER status is an important feature that greatly determines the patients’ treatment and, sometimes, fate. Specifically, it was shown that ILK attenuation either via silencing or through pharmacological inhibition leads to hyperphosphorylation of ERα while ILK overexpression promotes cell migration [44].

Another interesting interaction of ILK is the one with Hypoxia-inducible Factor (HIF) with which it co-localizes in human BC tissues [45] which explains why ILK expression and kinase activity are hypoxia-induced. Moreover, ILK in turn stimulates HIF-1α expression through cell type- and cell context-dependent pathways [46] accounting for the hypoxia effects observed on Akt, mTOR, and GSK3β phosphorylation, and ultimately leading to EMT.

## ILK in BC: the use of pharmacological inhibitors

To explore ILK’s role in BC, a number of studies were performed using pharmacological inhibition of ILK (Table 1). Troussard, et al., (2006) [47] attempted to show that ILK is a critical regulator of BC cell survival through the PKB/Akt pathway by using QLT0267, which resulted in the inhibition of PKB/Akt Ser473 phosphorylation, stimulation of apoptosis, and decreased mTOR expression in human BC cells. This inhibitor was very drastic in deactivating ILK, which led to MDA-MB-231 cancer cell apoptosis. Similarly, QLT0267 dramatically inhibited cell invasion and completely abolished signaling via Akt, ERK, and c-Jun phosphorylation in MDA-MB-231 cells, resulting in uPA down-regulation [48].

Moreover, Kalra et al. [49], showed that when QLT0267 inhibitor was combined with other therapeutic agents, such as docetaxel (Dt), the result was even more impressive. Also, QLT0267/Dt combination was shown to increase cancer cell apoptosis [49]. Furthermore, in nude mice, QLTO267/Dt combination inhibited cancer cell migration ability [50]. In another study [51], Human Epidermal growth factor Receptor 2/neu (Her2/neu) signaling was evaluated in six Her2/neu(+) BC cell lines (LCC6Her2, MCF7Her2, SKBR3, ΒΤ474, JIMT-1, and KPL-4) that were treated with ILK inhibitor QLT0267 and resulted in a 32–87% suppression of total Her2/neu protein in these cells [51]. Thus, ILK silencing resulted in a transient decrease in Ρ-ΑΚΤser473, which was not related to 22/neu downregulation. Attenuation of ILK expression was also associated with decreases in Y-box binding protein-1 (YB-1) expression, a known transcriptional regulator of Her2/neu expression [51] while ILK overexpression was associated with a fourfold increase in the YB-1 expression. Therefore, it was suggested that since ILK regulates the expression of Her2/neu and YB-1, the use of ILK inhibitors could be beneficial for the treatment of aggressive Her2/neu(+) BC tumors.

Using another ILK inhibitor, known as T315, in MDA-MB-468 BC cells which exhibit elevated expression of ILK, Lee, et al. [52] demonstrated that ILK inhibition resulted in reduced phosphorylation of Ser473-Akt. ILK was also shown to form complex with Rictor that was easily disrupted by ILK inhibitor T315 and they finally showed that ILK inhibition either by T315 or through siRNA-mediated silencing suppressed EMT in these cells, suggesting a metastasis-promoting function of ILK in BC cells.

## ILK in BC: cancer stem cells (CSC)

Interestingly, ILK has been shown to be involved in the development of BC stem-like cells (CSC), which are tumor-generating, having the capacity to produce tumors through stem cell self-renewal and differentiation (Table 1). Such cells exist in tumors as a separate low-density population and cause regression and metastasis, provoking new tumors [53]. Especially BC tumors are considered to be stiff and hypoxic and have been shown to promote the development of BC CSC through modulation of ILK [54]. More specifically, ILK depletion through short hairpin RNA (shRNA)-mediated silencing was shown to block the hypoxia-dependent acquisition of CSC marker expression and behavior, whereas ectopic expression of ILK stimulated CSC development. Notably, this association between ILK and CSC markers was verified in human BC samples [54].

In a similar approach, Hsu, et al. [55] investigated the signaling of interleukin-6 (IL-6) and Notch, considering that they are important regulators of BC CSC. They investigated the role of ILK in regulating IL-6-driven Notch 1 activation and the ability to target breast CSCs through ILK inhibition. Luciferase assays were used to evaluate the regulation of IL-6-driven Notch1 activation by ILK in IL-6 (MDA-MB-231, SUM-159) and in MCF-7 and MCF-7^IL−6^ cells. Results showed that inhibition of ILK suppressed Notch1 activation while ILK silencing inhibited breast CSC-like properties *in vitro* and *in vivo* [55]. In subsequent studies, IL-6 was demonstrated to induce ILK expression via Ε2F1 upregulation, which, in turn, activates NF-κΒ signaling [56] in triple negative BC cells (MDA-MB-231 and MDA-MB-468) which are associated with more aggressive phenotypes, increased metastatic potential, and drug resistance.

## ILK in BC: conditional ILK knock-out and transgenic mice

Pontier, et al., (2010) [39] performed an *in vivo* study using conditional ILK knock-out mice under an MMTV promoter, that lacked ILK expression specifically from the mammary epithelium. Although mammary epithelial disruption of ILK had little impact on normal mammary gland development, loss of the ILK resulted in a significant inhibition of mammary tumor induction while inhibition of ILK function using an ILK inhibitor or siRNA-mediated silencing in ErbB2-expressing cell epithelium induced apoptosis and severely reduced cells’ *in vitro* invasive capacity [39]. Notably, the rare tumors that formed at some point were shown to have overcome this block in tumor induction by upregulation of ErB2 phosphorylation.

In the same year, Oloumi, et al., (2010) investigated the potential interaction between Wnt and ILK proteins during mammary tumor formation and progression [38]. To that regard, they established a transgenic mouse model that expressed both Wnt and ILK in mammary epithelial cells. A novel transgenic mouse model was generated by crossing two previously characterized mouse models, MMTV-Wnt1 and MMTV-ILK. They reported a significant acceleration in mammary tumor incidence and growth in the resulting MMTV-Wnt/ILK mice [38].

Finally, transgenic mice overexpressing ILK in the mammary epithelium, under the transcriptional control of MMTV were shown to develop a hyperplastic mammary phenotype, accompanied by phosphorylation of PKB/Akt, GSK-3β, and ERK [57].

## ILK in BC: human samples

Remarkably, a study conducted in China using BC patient samples obtained from 2005 to 2007 sheds more light on the role of ILK in BC. *ILK* expression was examined at the mRNA level in 64 BC patient samples while immunohistochemical analysis was also performed in 163 BC patient samples [58]. All patients undergoing BC surgery had not received radiation therapy or chemotherapy prior to surgery. Results showed that the relative *ILK* mRNA expression was significantly higher in BC tissues compared to normal adjacent tissues while it was also correlated with tumor size, grade, stage, ER status, and lymph node metastasis. Most importantly, all patients were monitored for 5 years after and the full clinical data were computer-registered. Analysis of the data using Kaplan-Meier analysis and Cox proportional hazard regression models revealed that there is a strong correlation between high ILK expression and reduced overall survival [58], further suggesting that ILK is an important molecular player of BC pathogenesis, progression, and prognosis.

Finally, in another study performed in 96 phyllodes BC, ILK was found to be highly expressed in the tumor and associated with increasing tumor grade. Moreover, analysis of EMT-related genes indicated decreased immunoreactivity of E-cadherin and β-catenin and increased expression of N-cadherin, vimentin, Snail, ZEB1, and Twist [59].

Based on the above, most studies performed so far, both *in vitro* and *in vivo,* suggest that ILK promotes tumor growth, EMT, and metastatic behavior of cells. There is one study though, which comes in complete contrast to this idea. Chen et al. [60] presented evidence that the chromosomal locus on which ILK maps (11p15.5) falls within a region on the short arm of chromosome 11 which is commonly lost and is considered as a potent event in the progression and metastasis of BC. Moreover, they showed that ILK was expressed in 20 normal breast tissue samples found adjacent to the tumor but it was significantly downregulated in the corresponding BC samples. They also overexpressed ILK in MDA-MB-435 cells which are highly metastatic and have a low endogenous ILK expression leading to growth-suppression activity both *in vitro* and *in vivo* [60]. The fact that these results are not in agreement with all previous studies can be explained by the small number of samples (20) and the use of a single cell line (MDA-MB-435) and further emphasizes the significance of tumor heterogeneity for cancer treatment.

## Conclusion

**Figure 3.**
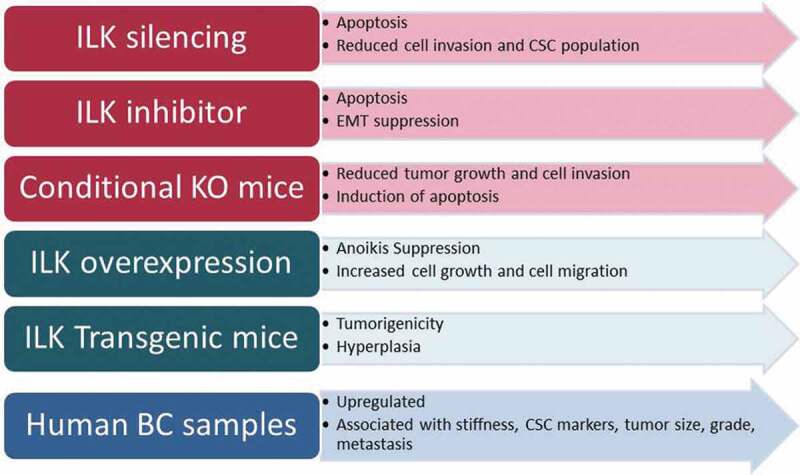
Summary of the studies performed *in vitro, in vivo* or in human BC samples regarding the role of ILK in BC using different experimental approaches.

In the current review, we analyzed all studies performed with regard to the role of ILK in BC which included *in vitro*, and *in vivo* studies as well as studies using human BC samples. Taking all the above into consideration, we could conclude all studies except for one show that ILK promotes cell growth and metastasis in BC. As shown in Figure 3, ILK attenuation either via silencing or via pharmaceutical inhibition leads to apoptosis and inhibits EMT, cell invasion, and CSC population, while the same pattern was observed in conditional knock-out animals. Consistent with these, ILK overexpression suppresses anoikis, promotes cell and tumor growth as well as metastasis *in vitro,* and favors tumorigenicity in transgenic animals. Finally, ILK is found upregulated in BC tumor samples and its expression is associated with grade, stage, ER and PR status and metastasis. Thus, ILK could certainly serve as a potent pharmaceutical target for better and more effective treatment of BC patients.

## Abbreviations

AP1: Activator protein 1
BC: Breast cancer
CSCs: Cancer stem cells
Dt: Docetaxel
ECM: extracellular matrix
EGF: Epidermal growth factor
EMT: Epithelial-Mesenchymal Transition
ER: estrogen receptor
ERK: extracellular signal regulated kinase
FA: focal adhesions
FAK: Focal adhesion kinase
Gsk3β: Glycogen synthase kinase 3 beta
HER2/neu: Human Epidermal growth factor Receptor2/neu
HIF: Hypoxia-inducible Factor
IL6: Interleukin 6
ILK: Integrin Linked Kinase
ILK-AP: ILK-associated protein
IRS1: Insulin Receptor Substrate-1
KD: kinase domain
MLC: myosin light chain
MMTV: mouse mammary tumor virus
mTOR: mammalian target of rapamycin
PARVA: α-parvin
PARVB: β-parvin
PDGF: Platelet Derived Growth Factor
PINCH-1: Particularly Interesting New Cysteine Histidine protein
PIP3: Phosphatidylinositol (3,4,5) triphosphate
P13k: Phosphoinositide 3- kinase
PP2C: Protein phosphatase 2C
PTEN: phosphatase and tensin homolog
RSU-1: Ras suppressor-1
TGFβ-1: Transforming growth factor beta 1
TNBC: Triple negative breast cancer
uPA: urokinase plasminogen activator
uPAR: urokinase plasminogen activator receptor
YAP: Yes associated protein 1
YB-1: Y box binding protein 1

## References

[1] Bray F, Ferlay J, Soerjomataram I, et al. Global cancer statistics 2018: GLOBOCAN estimates of incidence and mortality worldwide for 36 cancers in 185 countries. CA Cancer J Clin. 2018;68:394–424. 30207593 10.3322/caac.21492

[2] Chavez-MacGregor M, Mittendorf EA, Clarke CA, et al. Incorporating tumor characteristics to the american joint committee on cancer breast cancer staging system. Oncologist. 2017;22:1292–1300. 28592619 10.1634/theoncologist.2017-0116 PMC5679819

[3] Rakha EA, Reis-Filho JS, Baehner F, et al. Breast cancer prognostic classification in the molecular era: the role of histological grade. Breast Cancer Res. 2010;12:207. 20804570 10.1186/bcr2607 PMC2949637

[4] Cowell CF, Weigelt B, Sakr RA, et al. Progression from ductal carcinoma in situ to invasive breast cancer: revisited. Mol Oncol. 2013;7:859–869. 23890733 10.1016/j.molonc.2013.07.005 PMC5528459

[5] Pickup MW, Mouw JK, Weaver VM. The extracellular matrix modulates the hallmarks of cancer. EMBO Rep. 2014;15:1243–1253. 25381661 10.15252/embr.201439246 PMC4264927

[6] Wu C. ILK interactions. J Cell Sci. 2001;114:2549–2550. 11683382 10.1242/jcs.114.14.2549

[7] Widmaier M, Rognoni E, Radovanac K, et al. Integrin-linked kinase at a glance. J Cell Sci. 2012;125:1839–1843. 22637643 10.1242/jcs.093864

[8] Yoganathan N, Yee A, Zhang Z, et al. Integrin-linked kinase, a promising cancer therapeutic target: biochemical and biological properties. Pharmacol Ther. 2002;93:233–242. 12191615 10.1016/s0163-7258(02)00192-4

[9] Legate KR, Montanez E, Kudlacek O, et al. ILK, PINCH and parvin: the tIPP of integrin signalling. Nat Rev Mol Cell Biol. 2006;7:20–31. 16493410 10.1038/nrm1789

[10] McDonald PC, Fielding AB, Dedhar S. Integrin-linked kinase – essential roles in physiology and cancer biology. J Cell Sci. 2008;121:3121–3132. 18799788 10.1242/jcs.017996

[11] Delcommenne M, Tan C, Gray V, et al. Phosphoinositide-3-OH kinase-dependent regulation of glycogen synthase kinase 3 and protein kinase B/AKT by the integrin-linked kinase. Proc Natl Acad Sci U S A. 1998;95:11211–11216. 9736715 10.1073/pnas.95.19.11211 PMC21621

[12] Wu C. The PINCH-ILK-parvin complexes: assembly, functions and regulation. Biochim Biophys Acta. 2004;1692:55–62. 15246679 10.1016/j.bbamcr.2004.01.006

[13] Nikolopoulos SN, Turner CE. Integrin-linked kinase (ILK) binding to paxillin LD1 motif regulates ILK localization to focal adhesions. J Biol Chem. 2001;276:23499–23505. 11304546 10.1074/jbc.M102163200

[14] Wickstrom SA, Lange A, Montanez E, et al. The ILK/PINCH/parvin complex: the kinase is dead, long live the pseudokinase! Embo J. 2010;29:281–291. 20033063 10.1038/emboj.2009.376 PMC2824469

[15] Qian Y, Zhong X, Flynn DC, et al. ILK mediates actin filament rearrangements and cell migration and invasion through PI3K/Akt/Rac1 signaling. Oncogene. 2005;24:3154–3165. 15735674 10.1038/sj.onc.1208525

[16] Qu Y, Hao C, Xu J, et al. ILK promotes cell proliferation in breast cancer cells by activating the PI3K/Akt pathway. Mol Med Rep. 2017;16:5036–5042. 28791358 10.3892/mmr.2017.7180

[17] Attwell S, Mills J, Troussard A, et al. Integration of cell attachment, cytoskeletal localization, and signaling by integrin-linked kinase (ILK), CH-ILKBP, and the tumor suppressor PTEN. Mol Biol Cell. 2003;14:4813–4825. 12960424 10.1091/mbc.E03-05-0308 PMC284786

[18] Persad S, Attwell S, Gray V, et al. Inhibition of integrin-linked kinase (ILK) suppresses activation of protein kinase B/Akt and induces cell cycle arrest and apoptosis of PTEN-mutant prostate cancer cells. Proc Natl Acad Sci U S A. 2000;97:3207–3212. 10716737 10.1073/pnas.060579697 PMC16217

[19] Hannigan GE, McDonald PC, Walsh MP, et al. Integrin-linked kinase: not so ‘pseudo’ after all. Oncogene. 2011;30:4375–4385. 21602880 10.1038/onc.2011.177

[20] Serrano I, McDonald PC, Lock F, et al. Inactivation of the Hippo tumour suppressor pathway by integrin-linked kinase. Nat Commun. 2013;4:2976. 24356468 10.1038/ncomms3976 PMC3905719

[21] Sarbassov DD, Guertin DA, Ali SM, et al. Phosphorylation and regulation of Akt/PKB by the rictor-mTOR complex. Science. 2005;307:1098–1101. 15718470 10.1126/science.1106148

[22] McDonald PC, Oloumi A, Mills J, et al. Rictor and integrin-linked kinase interact and regulate Akt phosphorylation and cancer cell survival. Cancer Res. 2008;68:1618–1624. 18339839 10.1158/0008-5472.CAN-07-5869

[23] Troussard AA, Tan C, Yoganathan TN, et al. Cell-extracellular matrix interactions stimulate the AP-1 transcription factor in an integrin-linked kinase- and glycogen synthase kinase 3-dependent manner. Mol Cell Biol. 1999;19:7420–7427. 10523630 10.1128/mcb.19.11.7420 PMC84735

[24] Oloumi A, McPhee T, Dedhar S. Regulation of E-cadherin expression and beta-catenin/Tcf transcriptional activity by the integrin-linked kinase. Biochim Biophys Acta. 2004;1691:1–15. 15053919 10.1016/j.bbamcr.2003.12.002

[25] Deng JT, Sutherland C, Brautigan DL, et al. Phosphorylation of the myosin phosphatase inhibitors, CPI-17 and PHI-1, by integrin-linked kinase. Biochem J. 2002;367:517–524. 12144526 10.1042/BJ20020522 PMC1222907

[26] Qin J, Wu C. ILK: a pseudokinase in the center stage of cell-matrix adhesion and signaling. Curr Opin Cell Biol. 2012;24:607–613. 22763012 10.1016/j.ceb.2012.06.003 PMC3467332

[27] Hanks SK, Quinn AM, Hunter T. The protein kinase family: conserved features and deduced phylogeny of the catalytic domains. Science. 1988;241:42–52. 3291115 10.1126/science.3291115

[28] Boudeau J, Miranda-Saavedra D, Barton GJ, et al. Emerging roles of pseudokinases. Trends Cell Biol. 2006;16:443–452. 16879967 10.1016/j.tcb.2006.07.003

[29] Elad N, Volberg T, Patla I, et al. The role of integrin-linked kinase in the molecular architecture of focal adhesions. J Cell Sci. 2013;126:4099–4107. 23843624 10.1242/jcs.120295

[30] Horton ER, Humphries JD, James J, et al. The integrin adhesome network at a glance. J Cell Sci. 2016;129:4159–4163. 27799358 10.1242/jcs.192054 PMC5117201

[31] Dougherty GW, Chopp T, Qi SM, et al. The Ras suppressor Rsu-1 binds to the LIM 5 domain of the adaptor protein PINCH1 and participates in adhesion-related functions. Exp Cell Res. 2005;306:168–179. 15878342 10.1016/j.yexcr.2005.01.025

[32] Attwell S, Roskelley C, Dedhar S. The integrin-linked kinase (ILK) suppresses anoikis. Oncogene. 2000;19:3811–3815. 10949937 10.1038/sj.onc.1203711

[33] Gil D, Ciolczyk-Wierzbicka D, Dulinska-Litewka J, et al. The mechanism of contribution of integrin linked kinase (ILK) to epithelial-mesenchymal transition (EMT). Adv Enzyme Regul. 2011;51:195–207. 21035499 10.1016/j.advenzreg.2010.09.005

[34] Xing Y, Qi J, Deng S, et al. Small interfering RNA targeting ILK inhibits metastasis in human tongue cancer cells through repression of epithelial-to-mesenchymal transition. Exp Cell Res. 2013;319:2058–2072. 23707970 10.1016/j.yexcr.2013.05.014

[35] Serrano I, McDonald PC, Lock FE, et al. Role of the integrin-linked kinase (ILK)/Rictor complex in TGFbeta-1-induced epithelial-mesenchymal transition (EMT). Oncogene. 2013;32:50–60. 22310280 10.1038/onc.2012.30

[36] McPhee TR, McDonald PC, Oloumi A, et al. Integrin-linked kinase regulates E-cadherin expression through PARP-1. Dev Dyn. 2008;237:2737–2747. 18773488 10.1002/dvdy.21685

[37] Somasiri A, Howarth A, Goswami D, et al. Overexpression of the integrin-linked kinase mesenchymally transforms mammary epithelial cells. J Cell Sci. 2001;114:1125–1136. 11228156 10.1242/jcs.114.6.1125

[38] Oloumi A, Maidan M, Lock FE, et al. Cooperative signaling between Wnt1 and integrin-linked kinase induces accelerated breast tumor development. Breast Cancer Res. 2010;12:R38. 20565980 10.1186/bcr2592 PMC2917033

[39] Pontier SM, Huck L, White DE, et al. Integrin-linked kinase has a critical role in ErbB2 mammary tumor progression: implications for human breast cancer. Oncogene. 2010;29:3374–3385. 20305688 10.1038/onc.2010.86

[40] Yang J, Hou Y, Zhou M, et al. Twist induces epithelial-mesenchymal transition and cell motility in breast cancer via ITGB1-FAK/ILK signaling axis and its associated downstream network. Int J Biochem Cell Biol. 2016;71:62–71. 26693891 10.1016/j.biocel.2015.12.004

[41] Riaz A, Zeller KS, Johansson S. Receptor-specific mechanisms regulate phosphorylation of AKT at Ser473: role of RICTOR in beta1 integrin-mediated cell survival. PLoS One. 2012;7:e32081. 22384145 10.1371/journal.pone.0032081 PMC3284553

[42] Sepulveda JL, Wu C. The parvins. Cell Mol Life Sci. 2006;63:25–35. 16314921 10.1007/s00018-005-5355-1 PMC2792345

[43] Mongroo PS, Johnstone CN, Naruszewicz I, et al. Beta-parvin inhibits integrin-linked kinase signaling and is downregulated in breast cancer. Oncogene. 2004;23:8959–8970. 15467740 10.1038/sj.onc.1208112

[44] Acconcia F, Manavathi B, Mascarenhas J, et al. An inherent role of integrin-linked kinase-estrogen receptor alpha interaction in cell migration. Cancer Res. 2006;66:11030–11038. 17108142 10.1158/0008-5472.CAN-06-2676

[45] Abboud ER, Coffelt SB, Figueroa YG, et al. Integrin-linked kinase: a hypoxia-induced anti-apoptotic factor exploited by cancer cells. Int J Oncol. 2007;30:113–122. 17143519

[46] Chou CC, Chuang HC, Salunke SB, et al. A novel HIF-1alpha-integrin-linked kinase regulatory loop that facilitates hypoxia-induced HIF-1alpha expression and epithelial-mesenchymal transition in cancer cells. Oncotarget. 2015;6:8271–8285. 25821081 10.18632/oncotarget.3186 PMC4480751

[47] Troussard AA, McDonald PC, Wederell ED, et al. Preferential dependence of breast cancer cells versus normal cells on integrin-linked kinase for protein kinase B/Akt activation and cell survival. Cancer Res. 2006;66:393–403. 16397254 10.1158/0008-5472.CAN-05-2304

[48] Santos ND, Habibi G, Wang M, et al. Urokinase-type Plasminogen Activator (uPA) is Inhibited with QLT0267 a Small Molecule Targeting Integrin-linked Kinase (ILK). Transl Oncogenomics. 2007;2:85–97. 23645983 PMC3634623

[49] Kalra J, Warburton C, Fang K, et al. QLT0267, a small molecule inhibitor targeting integrin-linked kinase (ILK), and docetaxel can combine to produce synergistic interactions linked to enhanced cytotoxicity, reductions in P-AKT levels, altered F-actin architecture and improved treatment outcomes in an orthotopic breast cancer model. Breast Cancer Res. 2009;11:R25. 19409087 10.1186/bcr2252 PMC2716491

[50] Kalra J, Anantha M, Warburton C, et al. Validating the use of a luciferase labeled breast cancer cell line, MDA435LCC6, as a means to monitor tumor progression and to assess the therapeutic activity of an established anticancer drug, docetaxel (Dt) alone or in combination with the ILK inhibitor, QLT0267. Cancer Biol Ther. 2011;11:826–838. 21358264 10.4161/cbt.11.9.15183 PMC3100631

[51] Kalra J, Sutherland BW, Stratford AL, et al. Suppression of Her2/neu expression through ILK inhibition is regulated by a pathway involving TWIST and YB-1. Oncogene. 2010;29:6343–6356. 20838384 10.1038/onc.2010.366 PMC3007675

[52] Lee SL, Chou CC, Chuang HC, et al. Functional role of mTORC2 versus integrin-linked kinase in mediating Ser473-Akt phosphorylation in PTEN-negative prostate and breast cancer cell lines. PLoS One. 2013;8:e67149. 23840605 10.1371/journal.pone.0067149 PMC3686768

[53] Sreepadmanabh M, Toley BJ. Investigations into the cancer stem cell niche using in-vitro 3-D tumor models and microfluidics. Biotechnol Adv. 2018;36:1094–1110. 29559382 10.1016/j.biotechadv.2018.03.009

[54] Pang MF, Siedlik MJ, Han S, et al. Tissue stiffness and hypoxia modulate the integrin-linked kinase ILK to control breast cancer stem-like cells. Cancer Res. 2016;76:5277–5287. 27503933 10.1158/0008-5472.CAN-16-0579 PMC5026611

[55] Hsu EC, Kulp SK, Huang HL, et al. Function of integrin-linked kinase in modulating the stemness of IL-6-abundant breast cancer cells by regulating gamma-secretase-mediated notch1 activation in caveolae. Neoplasia. 2015;17:497–508. 26152358 10.1016/j.neo.2015.06.001 PMC4719004

[56] Hsu EC, Kulp SK, Huang HL, et al. Integrin-linked kinase as a novel molecular switch of the IL-6-NF-kappaB signaling loop in breast cancer. Carcinogenesis. 2016;37:430–442. 26905583 10.1093/carcin/bgw020 PMC5006214

[57] White DE, Cardiff RD, Dedhar S, et al. Mammary epithelial-specific expression of the integrin-linked kinase (ILK) results in the induction of mammary gland hyperplasias and tumors in transgenic mice. Oncogene. 2001;20:7064–7072. 11704830 10.1038/sj.onc.1204910

[58] Yang HJ, Zheng YB, Ji T, et al. Overexpression of ILK1 in breast cancer associates with poor prognosis. Tumour Biol. 2013;34:3933–3938. 23832543 10.1007/s13277-013-0981-y

[59] Akrida I, Nikou S, Gyftopoulos K, et al. Expression of EMT inducers integrin-linked kinase (ILK) and ZEB1 in phyllodes breast tumors is associated with aggressive phenotype. Histol Histopathol. 2018;33:937–949. 29608014 10.14670/HH-11-987

[60] Chen P, Shen WZ, Karnik P. Suppression of malignant growth of human breast cancer cells by ectopic expression of integrin-linked kinase. Int J Cancer. 2004;111:881–891. 15300800 10.1002/ijc.20340

